# Anharmonic Lattice Dynamics in Sodium Ion Conductors

**DOI:** 10.1021/acs.jpclett.2c00904

**Published:** 2022-06-22

**Authors:** Thomas M. Brenner, Manuel Grumet, Paul Till, Maor Asher, Wolfgang G. Zeier, David A. Egger, Omer Yaffe

**Affiliations:** † Department of Chemical and Biological Physics, Weizmann Institute of Science, Rehovot 76100, Israel; ‡ Department of Physics, Technical University of Munich, 85748 Garching, Germany; § Institute for Inorganic and Analytical Chemistry, University of Muenster, 48149 Münster, Germany

## Abstract

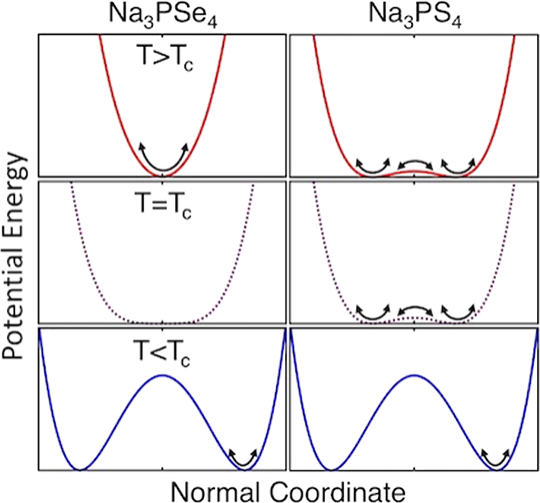

We employ terahertz-range temperature-dependent Raman spectroscopy and first-principles lattice dynamical calculations to show that the undoped sodium ion conductors Na_3_PS_4_ and isostructural Na_3_PSe_4_ both exhibit anharmonic lattice dynamics. The anharmonic effects in the compounds involve coupled host lattice–Na^+^ ion dynamics that drive the tetragonal-to-cubic phase transition in both cases, but with a qualitative difference in the anharmonic character of the transition. Na_3_PSe_4_ shows an almost purely displacive character with the soft modes disappearing in the cubic phase as the change in symmetry shifts these modes to the Raman-inactive Brillouin zone boundary. Na_3_PS_4_ instead shows an order–disorder character in the cubic phase, with the soft modes persisting through the phase transition and remaining Raman active in the cubic phase, violating Raman selection rules for that phase. Our findings highlight the important role of coupled host lattice–mobile ion dynamics in vibrational instabilities that are coincident with the exceptional conductivity of these Na^+^ ion conductors.

Solid-state ion conductors (SSICs) show great promise for enabling next-generation energy storage devices that are safer and more energy dense.^1^ The development of new, stable, and highly conductive SSICs requires a clear understanding of which material properties are essential to ion conductivity. Intensive research in recent years indicates that many highly conductive SSIC materials exhibit lattice dynamical phenomena consistent with strong anharmonicity.^2−12^ Anharmonicity refers to the coupling that occurs between vibrational normal modes (or phonons) of the lattice.^13,14^ Many materials exhibit mild anharmonicity that is expressed in thermal expansion, thermal conductivity, and finite phonon lifetimes. In contrast, SSICs are expected to exhibit strong anharmonicity because the process of hopping takes the mobile ion into a strongly anharmonic region of its potential energy, where it may couple to other vibrations present in the crystal.^6,15−17^

The strongly anharmonic behavior of SSIC materials was shown to have different expressions. For instance, plastic crystal phases and corresponding paddle wheel effects^18,19^ have been proposed in highly conductive, ionically bonded sulfide electrolytes^2−5^ and hydroborates.^10^ The decrease in activation energy caused by chemical, structural, or dynamic frustration suggests shallow, strongly anharmonic energy landscapes in the lattice dynamics of the corresponding compounds.^20,21^ Finally, relaxation phenomena tied to anharmonic effects have been observed in soft host lattices.^3,4,6^ In light of the latter, in a previous work on the structural dynamics of α-AgI (an archetypal SSIC), we proposed that host–lattice anharmonicity should be used as an experimental indicator in the design of new superionic conductors.^6^

In a recent important study, Gupta et al. used neutron scattering and molecular dynamics (MD) to establish the connection between anharmonic phonon dynamics and ionic conductivity in Na_3_PS_4_ with a high concentration of Na^+^ vacancies.^7^ Na_3_PS_4_ is the parent compound of Na_3–*x*_P_1–*x*_W_*x*_S_4_, a very high Na^+^ conductivity compound in which Na^+^ vacancies have been introduced through tungsten doping.^22^ They identified soft modes that stabilize the cubic phase. Furthermore, they demonstrated how these strongly anharmonic modes enable Na^+^ ions to hop along the minimum energy pathways.

However, neutron scattering is a costly experimental method that has many technical constraints.^24^ To implement anharmonicity as an indicator in material design, it is important to establish more accessible experimental characterization tools. To that end, Raman scattering spectroscopy is a very promising table-top technique that benefits from the ease of manipulating and detecting visible light with modern optics and microscopy. Therefore, it is useful even for very small sample sizes and/or weights and can be measured over a wide range of temperatures and pressures. As such, it is ideal for large throughput and spatial mapping with diffraction-limited resolution. Importantly, in recent years we demonstrated that terahertz-range Raman scattering combined with first-principles computations is very effective in elucidating the atomic-scale mechanisms that lead to anharmonic motion in solids.^6,25−28^

In this study, we investigate the temperature-dependent lattice dynamics of the foundational sodium ion conductors Na_3_PS_4_ and Na_3_PSe_4_ through terahertz-range Raman scattering and first-principles calculations. In both compounds, we clearly identify anharmonic vibrational modes involving coupled host lattice–Na^+^ ion motion that drive the tetragonal-to-cubic (*t-c*) phase transition, as reported previously in Na_3_PS_4_.^7,31^ Moreover, we demonstrate regime-crossing tunability of the material’s anharmonic character through the conceptually simple homovalent substitution of S for Se. While the phase transition is displacive in Na_3_PSe_4_, Na_3_PS_4_ exhibits dynamic symmetry breaking in a phase that is cubic only on average. Our findings demonstrate that the anharmonic lattice dynamics of SSICs can exhibit different underlying mechanisms even for seemingly minor substitutional changes.

Stoichiometric Na_3_PS_4_ and Na_3_PSe_4_ are known to take on tetragonal and cubic phases depending on the temperature (see Figure 1), with Na_3_PS_4_ having recently also been discovered to possess a plastic polymorph.^5,29,31−33^ The cubic structure (space group *I*4̅3*m*, *T*_*d*_^3^, #217) is composed of PCh_4_^3–^ (Ch = chalcogen) tetrahedra arranged on a BCC lattice with P atoms at BCC lattice sites and Na^+^ ions located at face centers and edges of the BCC cube. In the tetragonal structure (space group *P*4̅2_1_*c*, *D*_2*d*_^4^, #114) the PCh_4_^3–^ tetrahedra are tilted about the crystallographic *c*-axis while a subset of the Na^+^ ions are offset along the *c*-axis above and below their positions in the cubic phase.

**Figure 1:**
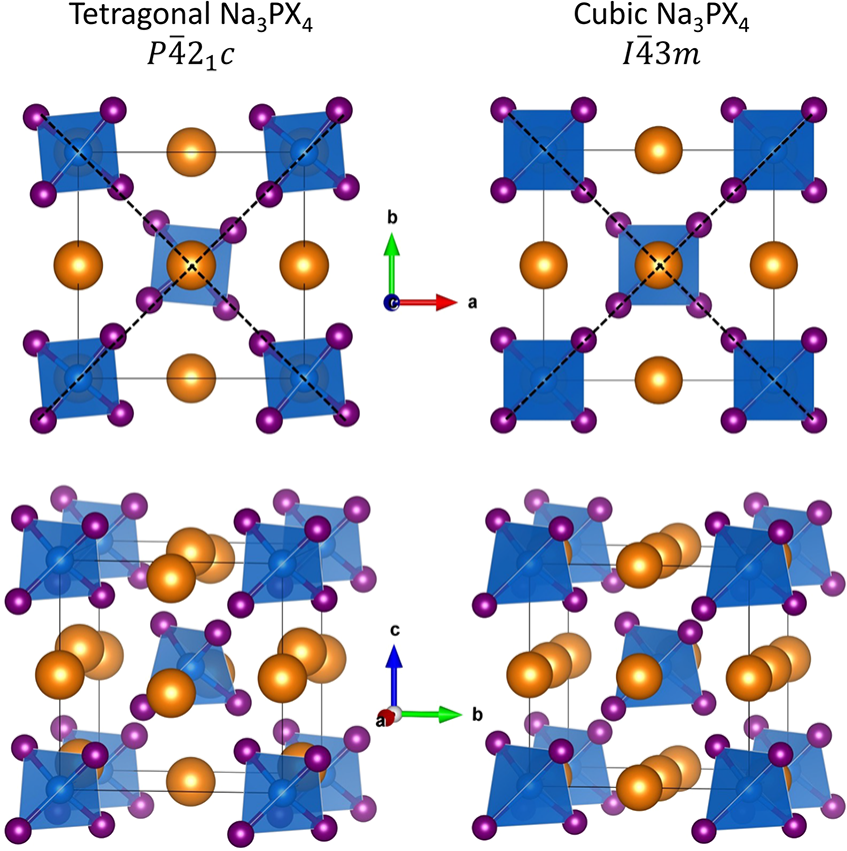
Schematic representation of the tetragonal^29^ and cubic^30^ structures of the Na_3_PCh_4_ (Ch = S or Se) crystal system. Na^+^ ions are colored orange, P atoms blue (and within blue-shaded PCh_4_^3–^ tetrahedra), and Ch atoms purple. The black dotted lines indicate the presence (*t* phase) or absence (*c* phase) of tetrahedral tilting about the *c*-axis. The offset of Na^+^ ions along the *c*-axis in the tetragonal compared to the cubic phase can be seen in the bottom panel.

Figure 2a shows the Raman spectra of Na_3_PSe_4_ and Na_3_PS_4_, normalized to the maximum intensity mode, throughout the temperature range encompassing the tetragonal and cubic phases of each compound. Both materials show a similar set of features numbered in bold for the lowest-temperature measurement as follows: a pair of peaks at a very high frequency (1), a sharp and intense single dominant peak (2), a group of intermediate-frequency modes (3), and a pair of very low-frequency modes (4). First-principles calculations of the Raman spectra, based on density functional theory (DFT) and the harmonic approximation (see Methods), find a set of features similar to those from experiment for both compounds (see Figure S1).

**Figure 2:**
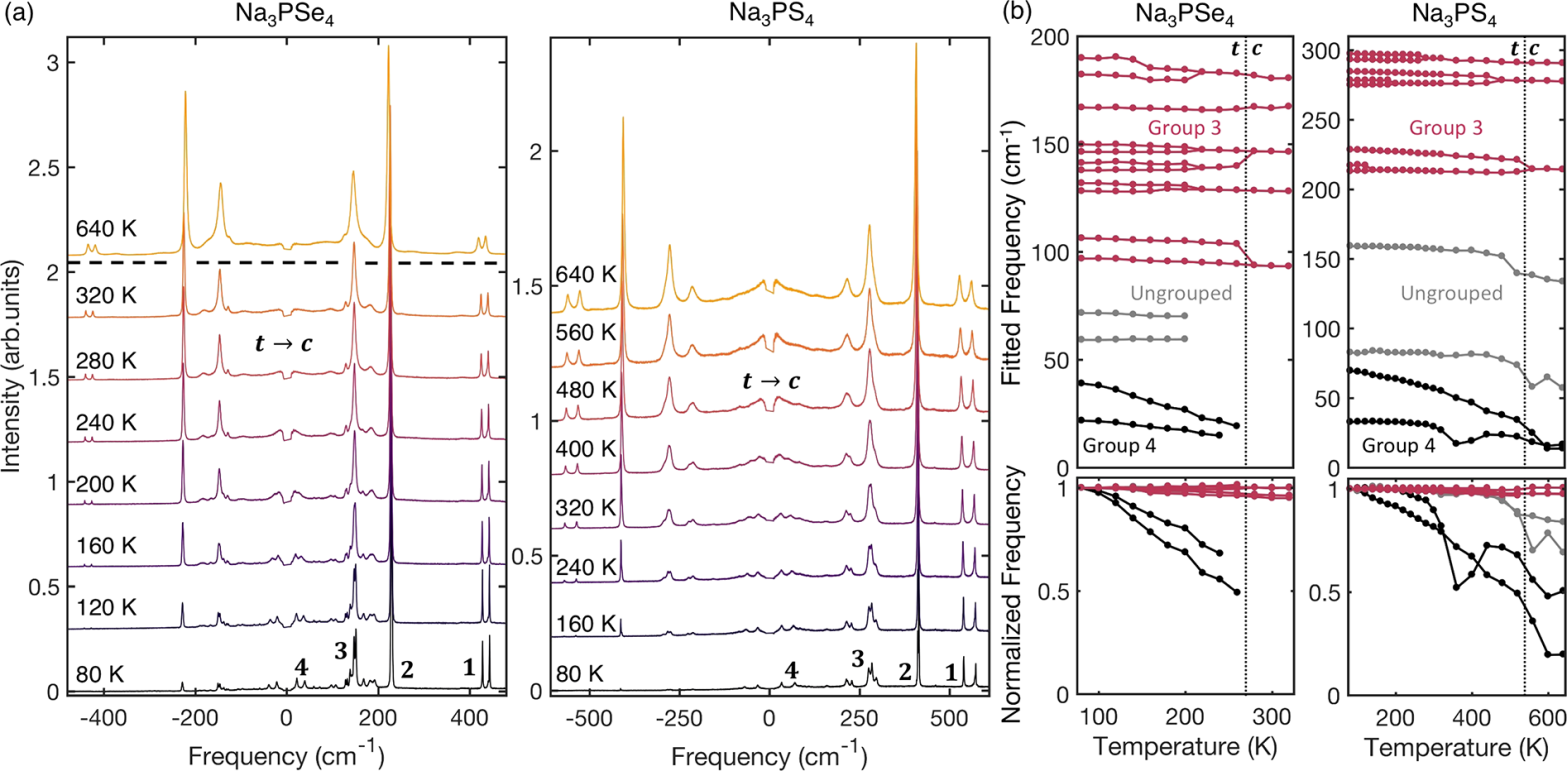
(a) Selected Raman spectra as a function of temperature for Na_3_PSe_4_ (between 80 and 320 K) and Na_3_PS_4_ (between 80 and 640 K), covering the *t-c* phase transition in both cases (see the *t-c* label). The bold numbers 1–4 mark groups of features shared by both materials. (b) Fit-derived frequency as a function of temperature (top) for the peaks in groups 3 (red) and 4 (black) for Na_3_PSe_4_ (left) and Na_3_PS_4_ (right). The *t-c* transition is marked by a dashed line. The normalized frequency plots (bottom) are the same as the top panels but show the fractional change in frequency. The peaks in group 4 (black) show anomalously strong shifts in relative frequency compared to the other modes in both compounds.

To quantify changes in the experimental spectra with temperature, we fit each spectrum with a multi-Lorentz oscillator fit (see Methods) to extract each peak’s temperature-dependent frequency. It was found that groups 1 and 2 show very little change with temperature, whereas the peaks of group 3 (Figure 2b) gradually merge as the temperature is increased toward the *t-c* transition. We defined the temperature of the *t-c* transition (*T*_c_) as occurring after the last peak has merged. This occurred between 260 and 280 K and between 520 and 560 K in Na_3_PSe_4_ and Na_3_PS_4_, respectively, both in agreement with X-ray diffraction measurements.^5,29,31,32,34^

The peaks of group 4 exhibit a notable temperature dependence (Figure 2b). As the temperature is increased, the frequency of these modes decreases much more quickly than for any of the other modes (bottom panels in Figure 2b), approaching zero frequency as *T*_c_ is approached. We investigate the mechanisms underlying the evolution of these modes by inspecting the temperature dependence of the low-frequency region of the Raman spectra (see Figure 3). All of the spectra in this figure have been normalized by the integrated intensity of the group 2 peak (see Figure 2) to enable comparison of the relative intensity to that of this peak. At 80 K, the peaks of group 4 appear to be sharp and well-resolved. As the temperature is increased, the peaks red-shift and broaden, eventually merging near the transition so the two peaks can no longer be distinguished. Above the *t-c* transition, the behavior of the two materials diverges. In Na_3_PSe_4_, the relative intensity of the group 4 feature decreases compared to that of the group 2 peak and the peak becomes broad and flat. This behavior persists up to 640 K, the highest temperature measured. In Na_3_PS_4_, the group 4 feature merges into one peak whose intensity relative to the group 2 peak remains relatively constant. The differing behavior of Na_3_PSe_4_ and Na_3_PS_4_ above the phase transition is a sign their structural dynamics are fundamentally different in character, as discussed further below.

**Figure 3:**
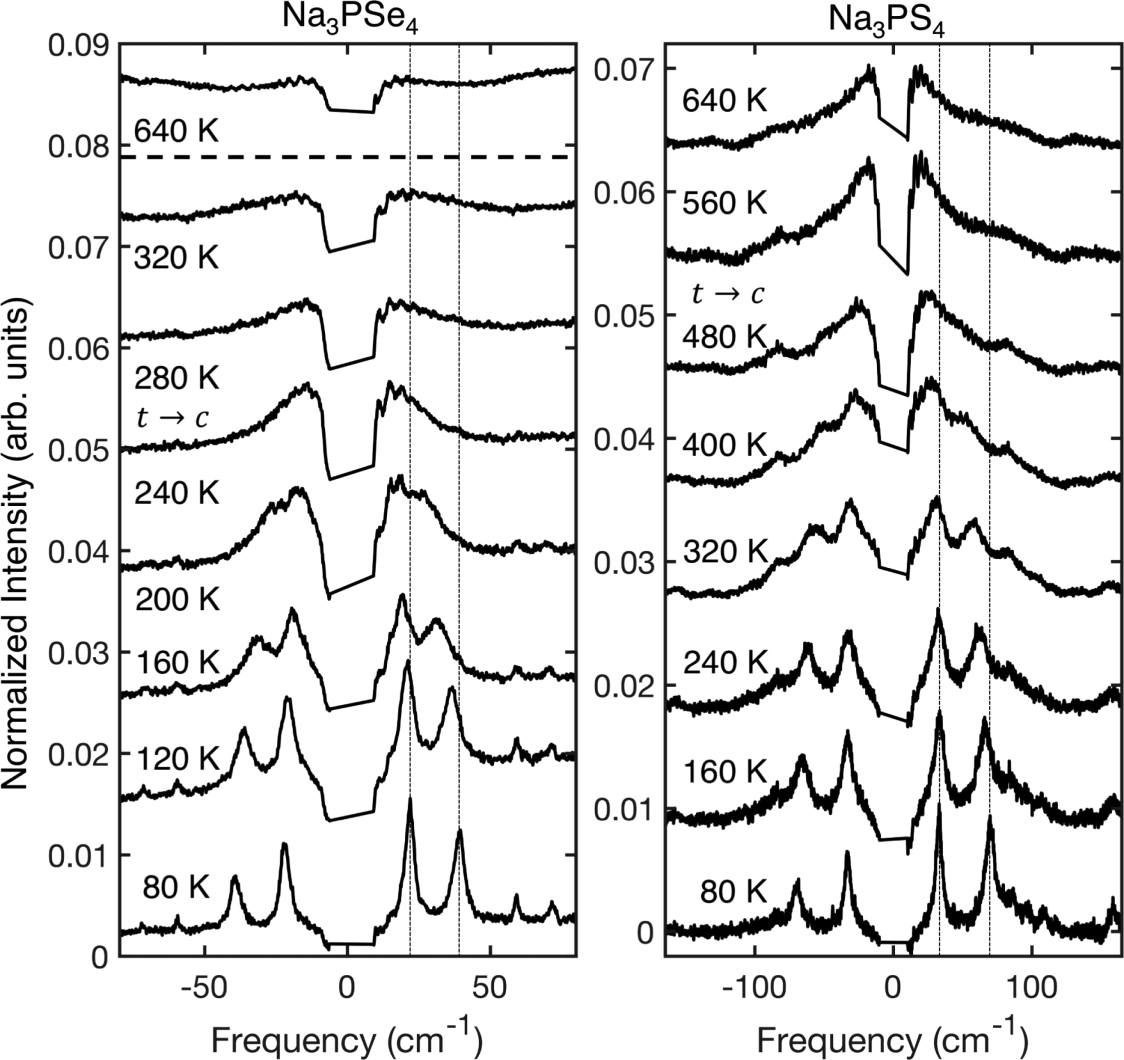
Low-frequency region of the Raman spectra as a function of temperature, covering the *t-c* phase transition (indicated in the figure) for Na_3_PSe_4_ and Na_3_PS_4_. This region contains two soft modes that shift toward 0 cm^–1^ as the temperature is increased. The two dashed vertical lines mark the location of the two soft modes at 80 K and serve as a guide to the eye for observing the shifts of each peak. The spectrum of Na_3_PSe_4_ at 640 K is included for direct temperature comparison to that of Na_3_PS_4_. All spectra have been normalized by the integrated intensity of the peak in group 2 to enable comparison of the relative intensity to that of this peak.

The behavior of the group 4 peaks can be explained by considering that they are soft modes that drive a displacive phase transition. Famprikis et al. and Gupta et al. also observed that the *t-c* transition in Na_3_PS_4_ is driven by a soft mode,^7,31^ and we additionally observe here that Na_3_PSe_4_ displays the same phenomenon. In a displacive phase transition, a gradual shift (displacement) of the atoms with temperature is driven by anharmonic interactions between the soft mode and other vibrations excited in the crystal at a given temperature. This eventually leads to a discontinuous change in the symmetry of the structure at the phase transition, when the atoms arrive at the positions and symmetry of the new crystal structure.^13,35−41^ Simultaneously, the frequency of the soft mode reaches zero as the phase transition is approached from temperatures both above and below the transition. At the transition temperature, the oscillatory motions of the atoms involved become a stationary distortion of the structure resulting in the new symmetry. Because of the symmetry changes involved in the transition, the soft mode usually appears as a single mode in the high-symmetry phase and as a pair of modes due to broken degeneracy in the low-symmetry phase. The group 4 peaks have the characteristics of the low-symmetry phase soft mode pair. The fact that the full decay to zero frequency is not observed here is due to the non-idealities in the real material system and instrument limits.

Above the phase transition temperature, information about the crystal symmetry combined with the Raman selection rules shows that the soft mode pair is expected to merge into a single-frequency triply degenerate soft mode located at the Brillouin zone boundary, which is not Raman active. Indeed, the DFT-calculated phonon dispersion of the cubic phase of both materials shows a lattice instability of a triply degenerate phonon at the Brillouin zone boundary (Figure S2), which cannot be accessed by Raman spectroscopy. The expected disappearance of the soft modes is observed in our experiments in Na_3_PSe_4_ but not in Na_3_PS_4_ (see Figure 3), again emphasizing their differing structural dynamical character.

Next, we extract the soft mode eigenvectors to examine if the process involves motion of the Na^+^ mobile ion. Keeping in mind that our 0 K phonon calculations do not account for any disorder or anharmoncity occurring at higher temperature, we can identify the soft modes in our DFT-computed Raman spectra of the low-temperature tetragonal phase (Figure S1) by their frequency and symmetry. These modes are expected to be the two lowest-frequency optical modes with single- and double-degeneracy symmetries (to combine into a triply degenerate mode). Indeed, we find such a pair of modes in computational spectra of both materials (Table S1). In Figure 4, we show the DFT-extracted eigenvectors of these modes for Na_3_PS_4_, with those of Na_3_PSe_4_ found in Figure S3. Interestingly, we find that there is coupling of mobile ion (Na^+^) and host lattice dynamics because the soft mode eigenvectors in both compounds exhibit a combination of tetrahedral tilting and Na^+^ translation with *A*_1_ (high-frequency mode) and *E* (low-frequency mode) vibrational symmetry (Table S1). For Na_3_PS_4_, this is in agreement with the results of Famprikis et al. and Gupta et al.^7,31^

**Figure 4:**
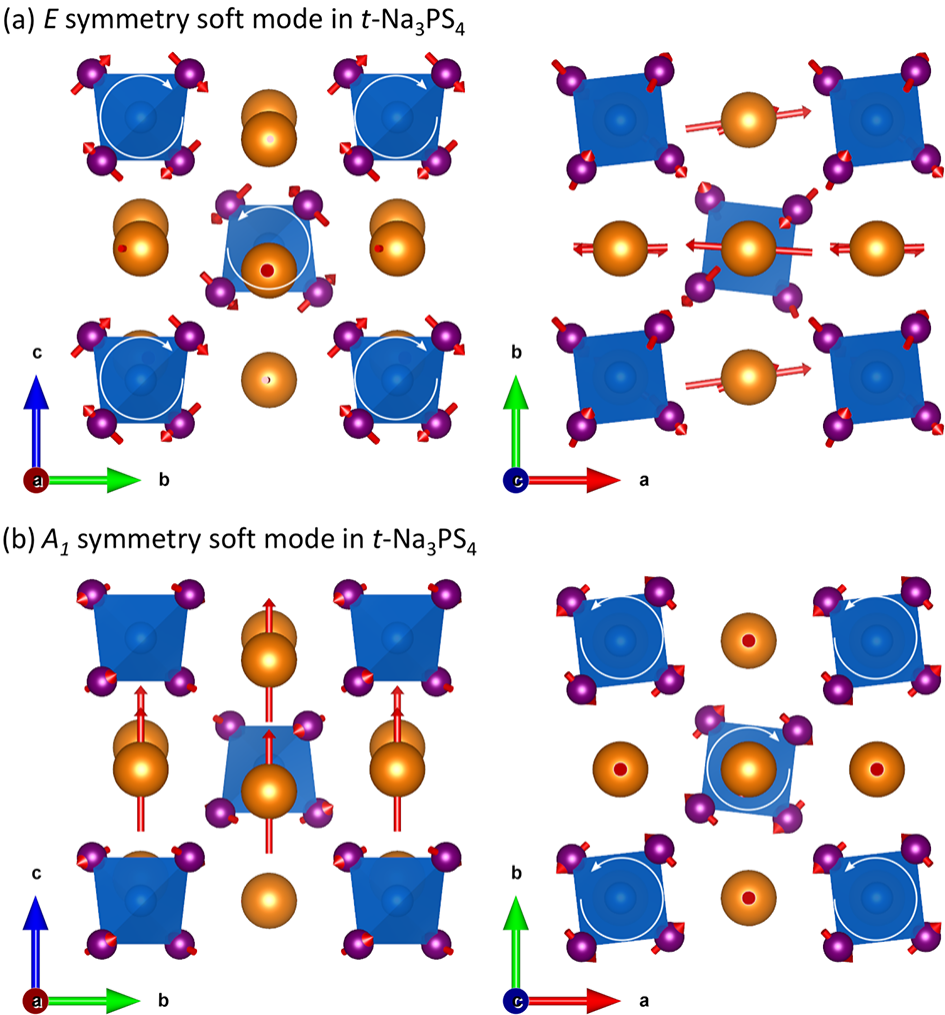
DFT-computed eigenvectors of the two soft modes identified from Raman spectroscopy in Na_3_PS_4_. In both cases, the mode with wavevector **q**∥*c* is shown, though other **q** directions show nearly identical eigenvectors. (a) The mode of *E* symmetry corresponds to the lower-frequency soft mode. This mode is doubly degenerate, and only one of the two modes is shown. It involves rotation of the PS_4_^3–^ tetrahedra about the *a*-axis (left panel, white arrows) and Na^+^ translation along the *a*-axis (right panel). The second degenerate mode has the same motion, but about the *b*-axis. (b) The mode of *A*_1_ symmetry corresponds to the higher-frequency soft mode. This mode involves rotation of the PS_4_^3–^ tetrahedra about the *c*-axis (right panel, white arrows) combined with Na^+^ translation along the *c*-axis (left panel). The findings for the Se material are qualitatively similar (see Figure S4).

The finding that collective tilting across the phase transition is mediated by the Na^+^ ions also allows us to rationalize that a tilting-like transition occurs in Na_3_PSe_4_ and Na_3_PS_4_, despite their tetrahedra being isolated, which is different compared to the cases of corner- or edge-sharing structures (e.g., perovskites). With these findings, we have established coupling between the motion of the mobile ion and host lattice within the soft mode lattice instability. Indeed, a number of prior works have discussed the role of coupled mobile ion–host lattice dynamics in ion conduction, including studies that have investigated Na_3_PS_4_.^2−4,6,7,10−12,18,19^

Having identified strong anharmonicity and its connections to Na^+^ dynamics in both compounds, we now compare the character of this anharmonicity between them. Interestingly, while these compounds both appear to exhibit a displacive phase transition, a closer inspection of the low-frequency spectral range in Figure 2a, shown in Figure 3, indicates that Na_3_PSe_4_ and Na_3_PS_4_ display differing anharmonic character. Above *T*_c_, the soft modes disappear in Na_3_PSe_4_, leaving a flat, broad feature that remains unchanged up to 640 K and is attributed to second-order Raman scattering. This is indeed what is expected for a purely displacive phase transition,^13^ because the cubic phase soft mode appears at the Brillouin zone boundary and therefore is Raman inactive (Figure S2). However, in Na_3_PS_4_, the soft modes persist into the cubic phase, merging into a broad peak centered at zero frequency.

The persistence of the soft mode feature in the cubic phase in Na_3_PS_4_, rather than its disappearance, indicates that the cubic structure observed in diffraction measurements is only a dynamically averaged structure while the instantaneous, local structure exhibits lower symmetry.^7,42^ Similar behavior has been observed in halide perovskites,^25,28,41^ and PbMO_3_ (M = Ti, Zr, or Hf) perovskites^43,44^ in which the appearance of first-order scattering in the cubic phase violates the Raman selection rules associated with the average structure. Following our finding that the soft mode shows Na^+^–host–lattice vibrational coupling, we attribute the persistent soft mode feature in Na_3_PS_4_ to relaxational motion along this soft mode eigenvector, in analogy to relaxational motion of the octahedral tilting modes found in halide perovskites.^28,41^ This assessment is supported by the strongly anharmonic thermal ellipsoids refined from synchrotron X-ray scattering experiments in the cubic phase^29^ and by refinements of pair distribution function (PDF) measurements.^31,42^ Thus, the cubic phase dynamically samples different tetrahedral tilting configurations. The disorder resulting from this dynamic symmetry breaking causes a violation of the Raman selection rules for the soft mode. In other words, for Na_3_PS_4_, our findings indicate a coexistence of displacive and order–disorder^45^ phase transition character.^35,46^ Another route to understanding relaxational motion along this eigenvector is to picture the atoms involved, both mobile ions and the host lattice, as sampling many configurations in a double-well potential along each of the crystallographic directions. Gupta et al. have established this mobile ion double well in Na_3_PS_4_ through nudged elastic band calculations.^7^

We note that we cannot confirm here any further selection rule violations in cubic Na_3_PS_4_ that might occur as a result of the lowered instantaneous symmetry, as previously proposed for the high-frequency (550 cm^–1^) modes.^31^ The splitting of these modes can be explained by LO/TO splitting^47^ that persists into the cubic phase (Figure S2).

This diverging anharmonic character of these two materials. The crystal chemistry of the two compounds is very similar, and both structures show strong covalent bonding within the PS_4_^3–^/PSe_4_^3–^ tetrahedra and ionic bonding between the Na^+^ ions and the tetrahedra. We note that the Shannon ionic radius of Se^2–^ (1.98 Å) is slightly larger than that of S^2–^ (1.84 Å), so size effects may play a role. The different lattice dynamical behaviors observed here suggests that tuning anharmonic effects in solids can be very subtle, with a simple homovalent substitution changing the lattice dynamics qualitatively.

Our findings indicate that coupled anharmonic motion of the mobile ion and host lattice is an important structural dynamical feature of this class of sodium ion conductors, which display two qualitatively different manifestations of this anharmonicity in Na_3_PSe_4_ versus Na_3_PS_4_. Gupta et al. have shown that this particular anharmonic motion may assist ion conduction in this material through coordination of the jump process with dynamic modification of the host lattice bottleneck, arising from the nature of the soft mode motion.^7^ This mechanism of conductivity enhancement does not require full rotary motion of the anions as in the paddle wheel effect and also affords a coordination of motion that the random spinning of anions does not. Because Na_3_PS_4_ displays a more extreme form of anharmonic lattice instability, it is reasonable to predict that it has the capacity to be a better ion conductor than Na_3_PSe_4_ at high Na^+^ vacancy concentrations. However, the lower *t-c* transition temperature of Na_3_PSe_4_ suggests that it is easier for this lattice to shift lattice configurations, which could indicate that the lattice is more amenable to ion hops when a high Na^+^ vacancy concentration is present. An additional important factor is the fact that the aliovalent doping and the correspondingly generated Na vacancies that give record high conductivity in these compounds^2−5^ can also affect qualitative changes in the behavior of this lattice instability, and this is an area that requires further research.

In conclusion, we combined terahertz-range Raman scattering and DFT calculations to compare the structural dynamics of the Na^+^ ion conductors Na_3_PS_4_ and Na_3_PSe_4_, whose doped counterparts have recently demonstrated record Na^+^ conductivities.^22,23^ These compounds are isostructural, and both compounds possess a *t-c* phase transition at higher temperatures. Anharmonicities due to a vibrational instability in the cubic structure drive the phase transition. In the tetragonal phase, both compounds show telltale soft mode behavior, which indicates the instability of a single normal mode is the source of the phase transition. Our computational findings show the soft modes involve the coupled motion of the mobile Na^+^ ion and the host lattice, where the Na^+^ ions mediate the tilting of the PCh_4_^3–^ tetrahedra. Importantly, the structural dynamics of their cubic phases have divergent character. Na_3_PSe_4_ shows almost purely displacive character with the soft modes disappearing in the cubic phase as the change in symmetry shifts these modes to the Raman-inactive Brillouin zone boundary. Na_3_PS_4_ instead shows order–disorder character in the cubic phase, with the soft modes persisting through the phase transition and remaining active in Raman in the cubic phase, violating Raman selection rules for that phase. This indicates the cubic phase of Na_3_PS_4_ is only cubic on average and actually samples different atomic configurations in real time. While the origin of the diverging anharmonic behaviors is not yet clear, it is important to note that this substitution of a homovalent atom to form an isostructural material has led to dramatically different structural dynamics. The anharmonicity in this material and its tunability with substitution may both play an important role in the high conductivities of the doped compound, and this is suggested as an important direction for further work.

## Methods

*Material Synthesis*. Na_3_PS_4_ and Na_3_PSe_4_ were synthesized by high-temperature ampule synthesis. All synthesis preparations were carried out in an Ar-filled glovebox, and ampules were dried under dynamic vacuum at 800 °C for 2 h to remove all traces of water. The starting materials Na_2_S (Sigma-Aldrich, 99.98%) and P_2_S_5_ (Sigma-Aldrich, 99%) for Na_3_PS_4_ and Na_2_Se (self-synthesized^32^ with an adjusted heating ramp of 3 °C/h), P (99.995% trace metal basis, ChemPur), and Se (99.5% trace metal basis, Alfa Aesar) in the case of Na_3_PSe_4_ were ground together in an agate mortar. The homogenized mixtures were pressed into pellets and placed in quartz ampules (12 mm inner diameter), and the ampules sealed under vacuum. Reactions were performed in a tube furnace at 500 °C for 20 h with a heating ramp of 30 °C/h. The obtained pellets were ground into powders and stored in a glovebox for further use.

*Raman Scattering*. We performed Raman measurements on a custom-built Raman system designed for low-frequency Raman and collection of both Stokes and anti-Stokes scattering by using two notch filters (Ondax). We used a 785 nm diode laser (Toptica XTRA II) at powers of 5 mW (Na_3_PS_4_) and 2 mW (Na_3_PSe_4_) focused on the sample with a 50× NIR objective (Nikon Plan Apo NIR-C 50×/0.42). Beam powers were chosen so that beam heating (as measured by the Stokes/anti-Stokes ratio) was undetectable above the noise level at 80 K, the lowest measured temperature. Due to the extreme sensitivity of both materials to air, moisture, and local beam damage when performing Raman under vacuum, the following steps were performed. For low-temperature measurements (80–320 K), a powder sample of the material was pressed flat onto a glass coverslip and loaded into an inert atmosphere chamber inside a nitrogen-filled glovebox. The chamber consisted of a stainless steel blank bottom and an optical window top that were sealed together with a KF-flange (copper gasket). The KF-flange seal ensured that this chamber remained sealed even when placed under high-vacuum conditions, keeping the sample in a gaseous atmosphere. For Na_3_PS_4_, nitrogen from the glovebox was used as the working gas. For Na_3_PSe_4_, the working gas was helium. This was achieved by first sealing the chamber with a rubber O-ring gasket and then transferring it to a glovebag where the atmosphere was exchanged for helium and then the chamber was sealed with a copper gasket. For high-temperature measurements (>373 K), Na_3_PS_4_ and Na_3_PSe_4_ were flame-sealed inside a glass capillary tube under argon. This was done due to the spontaneous vaporization of some element(s) of Na_3_PS_4_ at temperatures above ∼100 °C (373 K). The sealed capillary prevented release of any elemental vapors and enabled an equilibrium vapor concentration.

Raman measurements at low temperatures (80–320 K) were performed by mounting the inert atmosphere cell with the sample inside onto the coldfinger of a cryostat (Janis, ST-500). The cryostat was pumped to high vacuum before low-temperature measurements commenced. The temperature was controlled with a Lakeshore temperature controller (model 335) with liquid nitrogen as the coolant. The sample temperature was calibrated against the cryostat set temperature by measuring the temperature of the inert cell directly using a temperature gauge. The inert cell temperature was found to be <5 K higher than the cryostat set point for all temperatures. Raman measurements at high temperatures (300–640 K) were performed by placing the Na_3_PS_4_ powder capillary onto the stage of a Linkam Temperature Controlled Stage (THMS600). The temperature was controlled by the Link software. Due to the slight risk of sulfur-containing vapors in case of a capillary burst during heating, the following precautions must be taken. The room must be well-ventilated room. The minimum amount of sample possible should be used. The capillary thickness should be suitable to the goal temperature, and the Linkam should be purged with inert gas to slow any reactions that may occur after breakage. To ensure the compatibility of the high-temperature and low-temperature data sets, measurements were overlapped at temperatures of 300 and 320 K.

The peak widths, positions, and Stokes/anti-Stokes ratio were found to be in agreement for both methods. The Raman spectra of both Na_3_PS_4_ and Na_3_PSe_4_ displayed a weak but noticeable background, which extended thousands of wavenumbers beyond the region of the Raman spectrum on the Stokes side. This indicates the presence of fluorescence or phosphorescence, likely from defects. This background was removed by specifying regions without Raman scattering, fitting a polynomial to these regions, and then subtracting the fitted polynomial. These backgrounded spectra are displayed in the text. After background subtraction, the spectrum was fit with a multipeak model. Stokes scattering and anti-Stokes scattering were fit simultaneously to verify that all features indeed arise from Raman scattering and to verify that the temperature changes monotonically. For Na_3_PS_4_, a damped Lorentz oscillator model was successful in fitting the peaks for all temperatures. The damped Lorentz oscillator, rather than a Lorentzian, was required to capture the broad features at low wavenumbers where the Lorentzian approximation does not hold. For Na_3_PSe_4_, we observed that the peaks could not be fit by either a pure damped Lorentz oscillator or a pure Gaussian peak shape. A pseudo-Voigt peak shape composed of a linear combination of a damped Lorentz oscillator and a Gaussian was employed to model the features of this sample. This suggests the peaks of this sample are broadened by both lifetime- and disorder-induced broadening. The relative weight of the Lorentz oscillator increased with temperature, supporting this hypothesis. The fitted equation for both materials can be expressed as
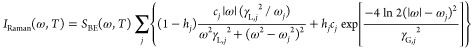
1where *I* is the Raman intensity, ω is the Raman shift, and *T* is the temperature. For the *j*th pseudo-Voigt peak, ω_*j*_ is the resonance frequency, γ_L,*j*_ and γ_G,*j*_ are the damping coefficients of the Lorentz oscillator and Gaussian components of the pseudo-Voigt, respectively, *c*_*j*_ is the intensity coefficient, and *h*_*j*_ is the relative weight of the Lorentz oscillator versus Gaussian character. An *h*_*j*_ value of 0 gives pure Lorentz oscillator character, and an *h*_*j*_ value of 1 gives pure Gaussian character. The sum of peaks is multiplied by the appropriate Bose–Einstein population factor [*S*_BE_(ω, *T*)] for Stokes and anti-Stokes scattering to account for the temperature dependence of the phonon populations. The lowest-temperature spectrum was fit first, and then the fit to the spectra at subsequent temperature steps was adapted from the previous step. When a pair of peaks could no longer be distinguished from a single peak, the fit to that feature was reduced to one peak.

Damped Lorentz oscillators oscillate at a frequency lower than their resonant frequency because of the damping. The fitted frequencies plotted in Figure 2b are the actual oscillation frequencies (ω_osc_) that are corrected by the damping coefficient to be
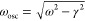
2

*First-Principles Calculations*. Calculations of zero-temperature phonon properties for both materials were performed using DFT. We applied the projector-augmented-wave method^48^ as implemented in the VASP code,^49,50^ with exchange correlation described by the Perdew–Burke–Ernzerhof (PBE) functional.^51^ In all calculations, the plane-wave energy cutoff was set to 350 eV and the energy threshold for electronic convergence was set to 10^–6^ eV.

Both the unit cell and the internal geometry of the two crystals were relaxed using the Gadget code,^52^ resulting in structures with forces smaller than ≈10^–3^ eV/Å. A Γ-centered 11 × 11 × 11 *k*-point grid was used in these relaxations. Furthermore, we geometrically constrained the crystal lattice vectors to maintain a cubic or tetragonal symmetry.

Phonon frequencies and eigenvectors were obtained by a finite-displacement method using the phonopy suite,^53^ with a supercell size of 128 atoms. To ensure the relatively tight settings that are required for phonon calculations, we used a 6 × 6 × 6 grid to compute force constants and an 11 × 11 × 11 grid to compute Born effective charges. Non-analytic corrections based on dipole–dipole interaction^54,55^ were included to correctly reproduce LO/TO splitting at the Γ point in the tetragonal and cubic phases.

Phonon-based Raman spectra were computed by performing polarizability calculations^56,57^ for each Raman-active phonon mode, using the phonopy-spectroscopy tool^58^ (see the Supporting Information). For these calculations, the *k*-point grid was reduced to 4 × 4 × 4, which we have verified to still guarantee sufficient numerical convergence. In the tetragonal phases, the **q**-direction dependence of the phonon modes near Γ that arises due to LO/TO splitting was accounted for using a spherical integration procedure based on seventh-order Lebedev–Laikov quadrature,^59^ which allowed us to obtain spectra that are spherically averaged over **q**.

## Supporting Information Available

Additional computational figures and tables, including computed Raman spectra, phonon dispersions, a table of Raman-active modes, and additional schematics of normal mode eigenvectors (PDF)
Transparent Peer Review report available (PDF)

## References

[ref1] Manthiram A.; Yu X.; Wang S. Lithium battery chemistries enabled by solid-state electrolytes. Nat. Rev. Mater. 2017, 2, 16103 10.1038/natrevmats.2016.103.

[ref2] Smith J. G.; Siegel D. J. Low-temperature paddlewheel effect in glassy solid electrolytes. Nat. Commun. 2020, 11, 1483 10.1038/s41467-020-15245-5. 32198363 PMC7083903

[ref3] Zhang Z.; Roy P.-N.; Li H.; Avdeev M.; Nazar L. F. Coupled Cation–Anion Dynamics Enhances Cation Mobility in Room-Temperature Superionic Solid-State Electrolytes. J. Am. Chem. Soc. 2019, 141, 19360 10.1021/jacs.9b09343. 31701751

[ref4] Zhang Z.; Li H.; Kaup K.; Zhou L.; Roy P.-N.; Nazar L. F. Targeting Superionic Conductivity by Turning on Anion Rotation at Room Temperature in Fast Ion Conductors. Matter 2020, 2, 1667 10.1016/j.matt.2020.04.027.

[ref5] Famprikis T.; Dawson J. A.; Fauth F.; Clemens O.; Suard E.; Fleutot B.; Courty M.; Chotard J.-N.; Islam M. S.; Masquelier C. A New Superionic Plastic Polymorph of the Na^+^ Conductor Na_3_PS_4_. ACS Mater. Lett. 2019, 1, 641 10.1021/acsmaterialslett.9b00322.

[ref6] Brenner T. M.; Gehrmann C.; Korobko R.; Livneh T.; Egger D. A.; Yaffe O. Anharmonic host-lattice dynamics enable fast ion conduction in superionic AgI. Phys. Rev. Materials 2020, 4, 115402 10.1103/PhysRevMaterials.4.115402.

[ref7] Gupta M. K.; Ding J.; Osti N. C.; Abernathy D. L.; Arnold W.; Wang H.; Hood Z.; Delaire O. Fast Na diffusion and anharmonic phonon dynamics in superionic Na_3_PS_4_. Energy Environ. Sci. 2021, 14, 6554 10.1039/D1EE01509E.

[ref8] Niedziela J. L.; Bansal D.; May A. F.; Ding J.; Lanigan-Atkins T.; Ehlers G.; Abernathy D. L.; Said A.; Delaire O. Selective breakdown of phonon quasiparticles across superionic transition in CuCrSe_2_. Nat. Phys. 2019, 15, 73 10.1038/s41567-018-0298-2.

[ref9] Ding J.; Niedziela J. L.; Bansal D.; Wang J.; He X.; May A. F.; Ehlers G.; Abernathy D. L.; Said A.; Alatas A.; Ren Y.; Arya G.; Delaire O. Anharmonic lattice dynamics and superionic transition in AgCrSe_2_. Proc. Natl. Acad. Sci. U.S.A. 2020, 117, 3930 10.1073/pnas.1913916117. 32029595 PMC7049114

[ref10] Kweon K. E.; Varley J. B.; Shea P.; Adelstein N.; Mehta P.; Heo T. W.; Udovic T. J.; Stavila V.; Wood B. C. Structural, Chemical, and Dynamical Frustration: Origins of Superionic Conductivity in closo-Borate Solid Electrolytes. Chem. Mater. 2017, 29, 9142 10.1021/acs.chemmater.7b02902.

[ref11] Adelstein N.; Wood B. C. Role of Dynamically Frustrated Bond Disorder in a Li^+^ Superionic Solid Electrolyte. Chem. Mater. 2016, 28, 7218 10.1021/acs.chemmater.6b00790.

[ref12] Duchêne L.; Lunghammer S.; Burankova T.; Liao W.-C.; Embs J. P.; Copéret C.; Wilkening H. M. R.; Remhof A.; Hagemann H.; Battaglia C. Ionic Conduction Mechanism in the Na_2_(B_12_H_12_)_0.5_(B_10_H_10_)_0.5_ closo-Borate Solid-State Electrolyte: Interplay of Disorder and Ion–Ion Interactions. Chem. Mater. 2019, 31, 3449 10.1021/acs.chemmater.9b00610.

[ref13] Dove M. T. Structure and dynamics: An atomic view of materials; Oxford University Press, 2003; Vol. 1.

[ref14] Califano S.; Schettino V.; Neto N. Lattice Dynamics of Molecular Crystals; Springer: Berlin, 1981; pp 1–40.

[ref15] Rice S. A. Dynamical Theory of Diffusion in Crystals. Phys. Rev. 1958, 112, 804 10.1103/PhysRev.112.804.

[ref16] Vineyard G. H. Frequency factors and isotope effects in solid state rate processes. J. Phys. Chem. Solids 1957, 3, 121 10.1016/0022-3697(57)90059-8.

[ref17] Salamon M. B., Ed. Physics of Superionic Conductors; Springer-Verlag: Berlin, 1979.

[ref18] Jansen M. Volume Effect or Paddle-Wheel Mechanism—Fast Alkali-Metal Ionic Conduction in Solids with Rotationally Disordered Complex Anions. Angew. Chem., Int. Ed. Engl. 1991, 30, 1547 10.1002/anie.199115471.

[ref19] Lundén A. Evidence for and against the paddle-wheel mechanism of ion transport in superionic sulphate phases. Solid State Commun. 1988, 65, 1237 10.1016/0038-1098(88)90930-1.

[ref20] Wood B. C.; Varley J. B.; Kweon K. E.; Shea P.; Hall A. T.; Grieder A.; Ward M.; Aguirre V. P.; Rigling D.; Lopez Ventura E.; Stancill C.; Adelstein N. Paradigms of frustration in superionic solid electrolytes. Philos. Trans. R. Soc., A 2021, 379, 20190467 10.1098/rsta.2019.0467. PMC8529417 34628943

[ref21] Di Stefano D.; Miglio A.; Robeyns K.; Filinchuk Y.; Lechartier M.; Senyshyn A.; Ishida H.; Spannenberger S.; Prutsch D.; Lunghammer S.; Rettenwander D.; Wilkening M.; Roling B.; Kato Y.; Hautier G. Superionic Diffusion through Frustrated Energy Landscape. Chem. 2019, 5, 2450 10.1016/j.chempr.2019.07.001.

[ref22] Fuchs T.; Culver S. P.; Till P.; Zeier W. G. Defect-Mediated Conductivity Enhancements in Na_3–*x*_Pn_1–*x*_*W*_*x*_S_4_ (Pn = P, Sb) Using Aliovalent Substitutions. ACS Energy Lett. 2020, 5, 146 10.1021/acsenergylett.9b02537.

[ref24] Abernathy D. L.; Stone M. B.; Loguillo M. J.; Lucas M. S.; Delaire O.; Tang X.; Lin J. Y. Y.; Fultz B. Design and operation of the wide angular-range chopper spectrometer ARCS at the Spallation Neutron Source. Rev. Sci. Instrum. 2012, 83, 015114 10.1063/1.3680104. 22299993

[ref25] Yaffe O.; Guo Y.; Tan L. Z.; Egger D. A.; Hull T.; Stoumpos C. C.; Zheng F.; Heinz T. F.; Kronik L.; Kanatzidis M. G.; Owen J. S.; Rappe A. M.; Pimenta M. A.; Brus L. E. Local Polar Fluctuations in Lead Halide Perovskite Crystals. Phys. Rev. Lett. 2017, 118, 136001 10.1103/PhysRevLett.118.136001. 28409968

[ref26] Asher M.; Angerer D.; Korobko R.; Diskin-Posner Y.; Egger D. A.; Yaffe O. Anharmonic Lattice Vibrations in Small-Molecule Organic Semiconductors. Adv. Mater. 2020, 32, 1908028 10.1002/adma.201908028. 32003507

[ref27] Sharma R.; et al. Lattice mode symmetry analysis of the orthorhombic phase of methylammonium lead iodide using polarized Raman. Physical Review Materials 2020, 4, 051601 10.1103/PhysRevMaterials.4.051601.

[ref28] Sharma R.; Dai Z.; Gao L.; Brenner T. M.; Yadgarov L.; Zhang J.; Rakita Y.; Korobko R.; Rappe A. M.; Yaffe O. Elucidating the atomistic origin of anharmonicity in tetragonal CH_3_NH_3_PbI_3_ with Raman scattering. Phys. Rev. Mater. 2020, 4, 092401 10.1103/PhysRevMaterials.4.092401.

[ref29] Nishimura S.-I.; Tanibata N.; Hayashi A.; Tatsumisago M.; Yamada A. The crystal structure and sodium disorder of high-temperature polymorph β-Na_3_PS_4_. J. Mater. Chem. A 2017, 5, 25025 10.1039/C7TA08391B.

[ref30] Bo S.-H.; Wang Y.; Kim J. C.; Richards W. D.; Ceder G. Computational and Experimental Investigations of Na-Ion Conduction in Cubic Na_3_PSe_4_. Chem. Mater. 2016, 28, 252 10.1021/acs.chemmater.5b04013.

[ref31] Famprikis T.; Bouyanfif H.; Canepa P.; Zbiri M.; Dawson J. A.; Suard E.; Fauth F.; Playford H. Y.; Dambournet D.; Borkiewicz O. J.; Courty M.; Clemens O.; Chotard J.-N.; Islam M. S.; Masquelier C. Insights into the Rich Polymorphism of the Na^+^ Ion Conductor Na_3_PS_4_ from the Perspective of Variable-Temperature Diffraction and Spectroscopy. Chem. Mater. 2021, 33, 5652 10.1021/acs.chemmater.1c01113. 34483480 PMC8411865

[ref32] Krauskopf T.; Pompe C.; Kraft M. A.; Zeier W. G. Influence of Lattice Dynamics on Na^+^ Transport in the Solid Electrolyte Na_3_PS_4–*x*_Se_*x*_. Chem. Mater. 2017, 29, 8859 10.1021/acs.chemmater.7b03474.

[ref33] Pompe C. Strukturchemie und elektrische Leitfähigkeiten von Natriumchalkogenometallaten. Ph.D. Thesis, Universität Regensburg, Regensburg, Germany, 2016.

[ref34] Krauskopf T.; Muy S.; Culver S. P.; Ohno S.; Delaire O.; Shao-Horn Y.; Zeier W. G. Comparing the Descriptors for Investigating the Influence of Lattice Dynamics on Ionic Transport Using the Superionic Conductor Na_3_PS_4–*x*_Se_*x*_. J. Am. Chem. Soc. 2018, 140, 14464 10.1021/jacs.8b09340. 30284822

[ref35] Dove M. T. Review: Theory of displacive phase transitions in minerals. Am. Mineral. 1997, 82, 213 10.2138/am-1997-3-401.

[ref36] Fultz B. Phase Transitions in Materials, 2nd ed.; Cambridge University Press, 2020; pp 482–510.

[ref37] Blinc R.; Zeks B. Soft Modes in Ferroelectrics and Antiferroelectrics; Elsevier: Amsterdam, 1974; p 317.

[ref38] Makarova I. P.; Misyul S. V.; Muradyan L. A.; Bovina A. F.; Simonov V. I.; Aleksandrov K. S. Anharmonic Thermal Atomic Vibrations in the Cubic Phase of Cs_2_NaNdCl_6_ Single Crystals. Physica Status Solidi B 1984, 121, 481–486. 10.1002/pssb.2221210205.

[ref39] Knudsen G. Soft mode and structural phase transition in the cubic elpasolite Cs_2_NaNdCl_6_. Solid State Commun. 1984, 49, 1045–1047. 10.1016/0038-1098(84)90419-8.

[ref40] Gorev M. V.; Misyul S. V.; Bovina A. F.; Iskornev I. M.; Kokov I. T.; Flerov I. N. Thermodynamic properties of elpasolites Cs_2_NaNdCl_6_ and Cs_2_NaPrCl_6_. Journal of Physics C: Solid State Physics 1986, 19, 2441 10.1088/0022-3719/19/14/010.

[ref41] Cohen A.; Brenner T. M.; Klarbring J.; Sharma R.; Fabini D. H.; Korobko R.; Nayak P. K.; Hellman O.; Yaffe O. Diverging Expressions of Anharmonicity in Halide Perovskites. Adv. Mater. 2022, 34, 2107932 10.1002/adma.202107932. 35076969

[ref42] Krauskopf T.; Culver S. P.; Zeier W. G. Local Tetragonal Structure of the Cubic Superionic Conductor Na_3_PS_4_. Inorg. Chem. 2018, 57, 4739 10.1021/acs.inorgchem.8b00458. 29613779

[ref43] Roleder K.; Jankowska-Sumara I.; Kugel G. E.; Maglione M.; Fontana M. D.; Dec J. Antiferroelectric and ferroelectric phase transitions of the displacive and order-disorder type in PbZrO_3_ and PbZr_1-x_TixO_3_ single crystals. Phase Transitions 2000, 71, 287–306. 10.1080/01411590008209310.

[ref44] Xu B.; Hellman O.; Bellaiche L. Order-disorder transition in the prototypical antiferroelectric PbZrO_3_. Phys. Rev. B 2019, 100, 020102 10.1103/PhysRevB.100.020102.

[ref45] “Order–disorder” is used here in the lattice dynamics sense^35,46^ rather than the alloy mixing sense.^36^

[ref46] Armstrong R. L. Displacive order-disorder crossover in perovskite and antifluorite crystals undergoing rotational phase transitions. Prog. Nucl. Magn. Reson. Spectrosc. 1989, 21, 151–173. 10.1016/0079-6565(89)80002-0.

[ref47] Yu P. Y.; Cardona M. Fundamentals of Semiconductors; Springer Berlin Heidelberg: Berlin, 2010.

[ref23] Hayashi A.; Masuzawa N.; Yubuchi S.; Tsuji F.; Hotehama C.; Sakuda A.; Tatsumisago M. A sodium-ion sulfide solid electrolyte with unprecedented conductivity at room temperature. Nat. Commun. 2019, 10, 5266 10.1038/s41467-019-13178-2. 31748566 PMC6868223

[ref48] Kresse G.; Joubert D. From Ultrasoft Pseudopotentials to the Projector Augmented-Wave Method. Phys. Rev. B 1999, 59, 1758 10.1103/PhysRevB.59.1758.

[ref49] Kresse G.; Furthmüller J. Efficiency of Ab-Initio Total Energy Calculations for Metals and Semiconductors Using a Plane-Wave Basis Set. Comput. Mater. Sci. 1996, 6, 15–50. 10.1016/0927-0256(96)00008-0. 9984901

[ref50] Kresse G.; Furthmüller J. Efficient Iterative Schemes for Ab Initio Total-Energy Calculations Using a Plane-Wave Basis Set. Phys. Rev. B 1996, 54, 11169 10.1103/PhysRevB.54.11169. 9984901

[ref51] Perdew J. P.; Burke K.; Ernzerhof M. Generalized Gradient Approximation Made Simple. Phys. Rev. Lett. 1996, 77, 3865–3868. 10.1103/PhysRevLett.77.3865. 10062328

[ref52] Bučko T.; Hafner J.; Ángyán J. G. Geometry Optimization of Periodic Systems Using Internal Coordinates. J. Chem. Phys. 2005, 122, 124508 10.1063/1.1864932. 15836398

[ref53] Togo A.; Tanaka I. First Principles Phonon Calculations in Materials Science. Scr. Mater. 2015, 108, 1–5. 10.1016/j.scriptamat.2015.07.021.

[ref54] Gonze X.; Charlier J.-C.; Allan D.; Teter M. Interatomic Force Constants from First Principles: The Case of α-Quartz. Phys. Rev. B 1994, 50, 13035–13038. 10.1103/physrevb.50.13035. 9975485

[ref55] Gonze X.; Lee C. Dynamical Matrices, Born Effective Charges, Dielectric Permittivity Tensors, and Interatomic Force Constants from Density-Functional Perturbation Theory. Phys. Rev. B 1997, 55, 10355–10368. 10.1103/PhysRevB.55.10355.

[ref56] Baroni S.; Resta R. Ab Initio Calculation of the Macroscopic Dielectric Constant in Silicon. Phys. Rev. B 1986, 33, 7017–7021. 10.1103/physrevb.33.7017. 9938028

[ref57] Gajdoš M.; Hummer K.; Kresse G.; Furthmüller J.; Bechstedt F. Linear Optical Properties in the Projector-Augmented Wave Methodology. Phys. Rev. B 2006, 73, 045112 10.1103/physrevb.73.045112.

[ref58] Skelton J. M.; Burton L. A.; Jackson A. J.; Oba F.; Parker S. C.; Walsh A. Lattice Dynamics of the Tin Sulphides SnS2, SnS and Sn2S3: Vibrational Spectra and Thermal Transport. Phys. Chem. Chem. Phys. 2017, 19, 12452–12465. 10.1039/C7CP01680H. 28470289 PMC5450010

[ref59] Popov M. N.; Spitaler J.; Veerapandiyan V. K.; Bousquet E.; Hlinka J.; Deluca M. Raman Spectra of Fine-Grained Materials from First Principles. npj Comput. Mater. 2020, 6, 121 10.1038/s41524-020-00395-3.

