# Supplementary material for: Anharmonic Lattice Dynamics in Sodium Ion Conductors
Source: J Phys Chem Lett. 2022 Jun 22;13(25):5938–45. doi: 10.1021/acs.jpclett.2c00904 (PMC9251760; doi:10.1021/acs.jpclett.2c00904)
Supplement: Supplementary file 1 — jz2c00904_si_001.pdf [file jz2c00904_si_001.pdf]

Anharmonic Lattice Dynamics in Sodium Ion Conductors

Thomas M. Brenner,[1] Manuel Grumet,[2] Paul Till,[3] Maor Asher,[1]
Wolfgang G. Zeier,[3] David A. Egger,[2] and Omer Yaffe[1,*]

[1]*Department of Chemical and Biological Physics,*
Weizmann Institute of Science, Rehovot 76100, Israel
[2]*Department of Physics, Technical University of Munich, 85748 Garching, Germany*
[3]*Institute for Inorganic and Analytical Chemistry,*
University of Muenster, Münster 48149, Germany

* omer.yaffe@weizmann.ac.il

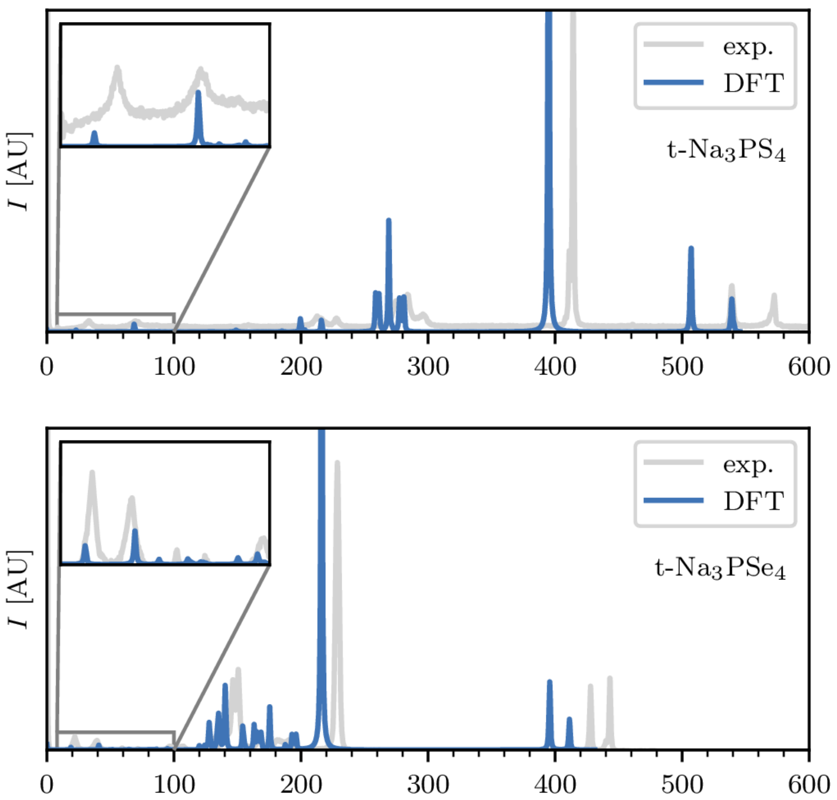

FIG. S1. DFT-calculated Raman spectra of tetragonal (t-) Na_3PSe_4 and Na_3PS_4 as compared to the experimental Raman spectra at 80K. The calculated spectra are weighted by the Bose-Einstein distribution at 80K to facilitate comparison. The inset shows a magnification of the soft mode region. While for both materials the overall qualitative agreement appears reasonable, a more profound discrepancy around 400 cm^{-1} between theoretical and experimental data remains, which might be due to limitations of the computational methods that were used.

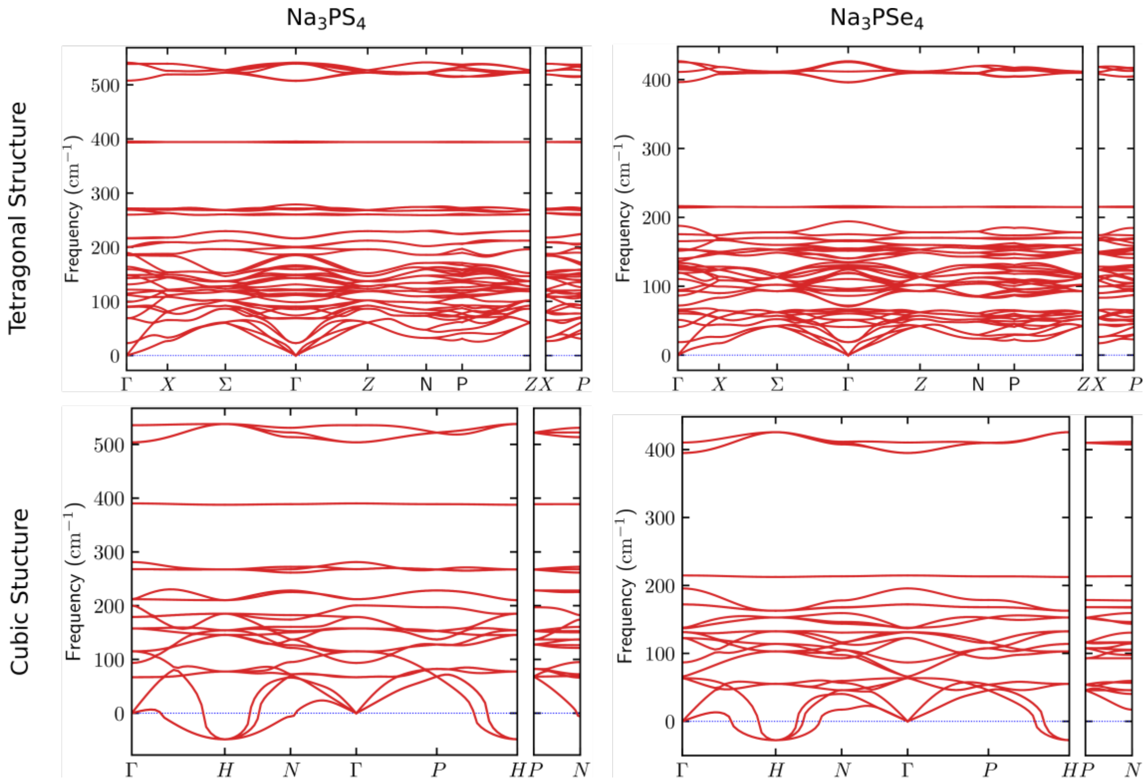

FIG. S2. DFT-calculated phonon dispersion curves for the tetragonal and cubic phases of Na_3PSe_4 and Na_3PS_4. Both compounds have a lattice instability at the H-point in the cubic phase, but no instability in the tetragonal phase.

TABLE S1. Table of DFT-computed Raman active modes for the tetragonal phase of Na_3PS_4 (left) and Na_3PSe_4 (right) - frequency, scattering cross-section, symmetry, and atoms involved. Modes with E symmetry are twofold degenerate.

ω [cm^{-1}]	I [AU]	χ	Atoms
-0.2	0.0	E	Na, P, S
-0.1	0.0	B_2	Na, P, S
22.8	1.0	E	Na, P, S
68.6	7.8	A_1	Na, S
69.2	1.1	E	Na, P, S
89.5	0.7	B_2	Na, P, S
90.0	0.9	E	Na, P, S
98.9	0.7	B_1	Na, P, S
111.6	0.7	E	Na, P, S
114.6	0.0	E	Na, P, S
121.7	0.0	A_2	Na, S
131.3	0.6	A_1	Na, S
142.7	0.2	E	Na, P, S
147.8	0.3	B_1	Na, P, S
148.7	3.6	E	Na, P, S
159.1	0.0	B_2	Na, P, S
163.9	0.1	A_2	Na, S
185.0	1.4	B_1	Na, P, S
186.0	1.4	E	Na, P, S
188.2	3.9	E	Na, P, S
199.5	17.0	A_1	Na, S
200.5	0.0	A_2	Na, S
216.0	14.8	B_1	Na, P, S
216.9	1.2	B_2	Na, P, S
259.9	0.8	B_2	Na, P, S
268.8	161.9	B_1	Na, P, S
269.1	269.2	E	Na, P, S
271.4	0.7	E	Na, P, S
393.0	0.0	A_2	Na, S
395.0	1174.1	A_1	Na, S
507.3	79.3	B_2	Na, P, S
522.6	159.8	E	Na, P, S
538.5	1.1	B_1	Na, P, S
540.4	1.6	E	Na, P, S

ω [cm^{-1}]	I [AU]	χ	Atoms
-0.7	0.0	E	Na, P, Se
-0.1	0.0	B_2	Na, P, Se
18.8	2.3	E	Na, P, Se
40.7	7.4	A_1	Na, Se
51.3	1.5	E	Na, P, Se
60.5	0.0	B_1	Na, P, Se
63.9	2.5	E	Na, P, Se
66.2	0.0	A_2	Na, Se
71.9	0.3	B_2	Na, P, Se
79.2	5.1	E	Na, P, Se
93.2	0.2	E	Na, P, Se
94.7	3.3	A_1	Na, Se
110.4	0.0	A_2	Na, Se
119.1	0.1	B_2	Na, P, Se
119.8	10.6	A_1	Na, P
124.1	10.1	B_1	Na, P, Se
125.7	4.7	E	Na, P, Se
127.5	84.3	E	Na, P, Se
130.5	0.1	B_1	Na, P, Se
133.7	0.0	A_2	Na, Se
136.3	201.8	B_2	Na, P, Se
140.1	189.5	E	Na, P, Se
151.3	0.4	B_1	Na, P, Se
152.8	0.6	B_1	Na, P, Se
154.0	100.2	E	Na, P, Se
165.2	19.1	B_2	Na, P, Se
170.3	127.7	E	Na, P, Se
181.5	252.7	E	Na, P, Se
214.0	0.0	A_2	Na, Se
216.2	2293.9	A_1	Na, Se
395.9	120.3	B_2	Na, P, Se
403.3	225.8	E	Na, P, Se
425.0	0.1	B_1	Na, P, Se
426.3	0.6	E	Na, P, Se

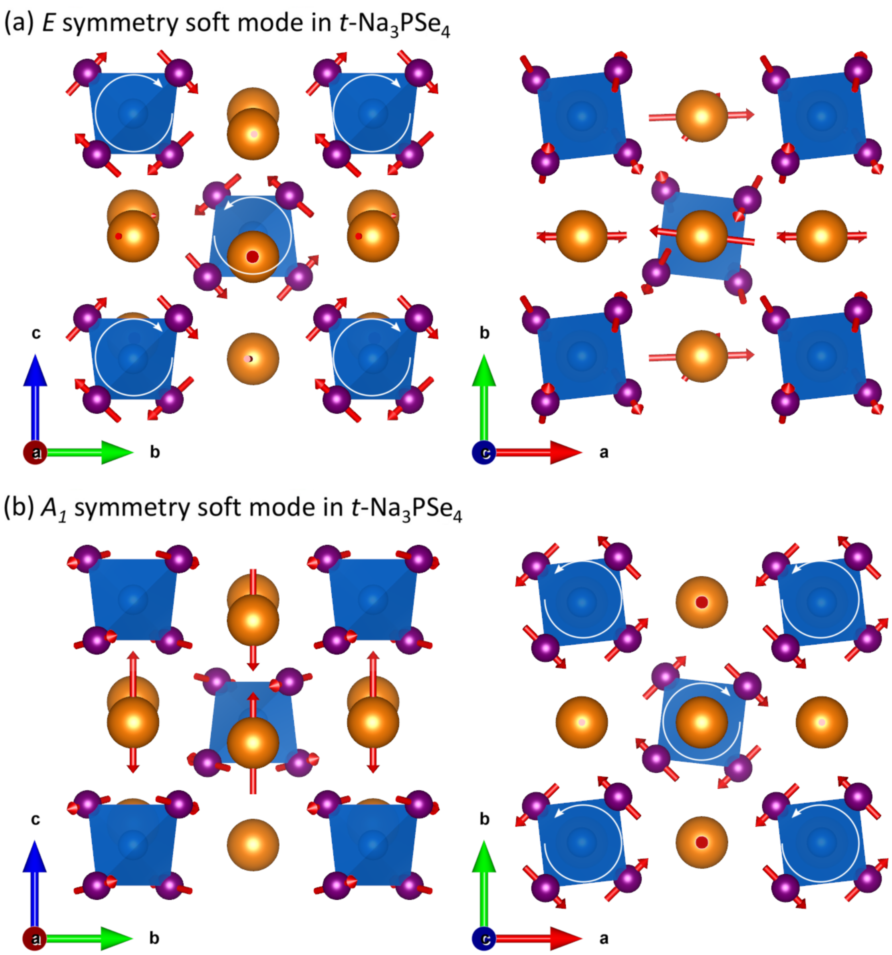

FIG. S3. Real-space representation of the soft mode eigenvectors in tetragonal (t-) Na_3PSe_4.
