# Supplementary material for: Anharmonic Lattice Dynamics in Sodium Ion Conductors
Source: J Phys Chem Lett. 2022 Jun 22;13(25):5938–45. doi: 10.1021/acs.jpclett.2c00904 (PMC9251760; doi:10.1021/acs.jpclett.2c00904)
Supplement: Supplementary file 2 — jz2c00904_si_002.pdf [file jz2c00904_si_002.pdf]

jz-2022-00904c.R1

Name: Peer Review Information for "Anharmonic Lattice Dynamics in Sodium Ion Conductors"

First Round of Reviewer Comments

Reviewer: 1

Comments to the Author

In their manuscript, Brenner et al. use Raman spectroscopy to identify a difference in the mode expression above the cubic transition in Na_3PS_4 and Na_3PSe_4. This difference is interpreted to mean that aspects of the local lattice symmetry of tetragonal Na_3PS_4 are retained above the transition and cubic Na_3PS_4 is cubic only as a dynamical average, whereas Na_3PSe_4 displays more conventional displacive phase transition character. The results are extremely interesting and provide significant value to the field, including a possibly new view of the nature of the cubic phase and its anharmonic coupling in Na_3PS_4.

I have a few comments:

1) The interpretation of elevated-temperature Raman spectra using zero-temperature DFT calculations is precarious, particularly for fast ion conductors. These materials have disordered cation configurations, yet the eigenvectors are associated with instantaneously idealized Wyckoff positions. The anharmonic changes to the phonon modes are also not well captured (unless molecular dynamics are used). Can the authors comment on the robustness of their conclusions in light of this?

2) The authors' interpretation seems to suggest that "nano" domains with different anharmonic tetragonal distortions persist in cubic Na_3PS_4, similar to dynamical averaging in perovskites. This is a very interesting conclusion, but it seems that another way to view these results is in terms of local cubic site distortions associated with a soft double-well potential in the cation potential energy landscape, which would manifest in the time-averaged cation probability density. Can the authors comment on this? It would be useful to more directly compare against MD studies of Na_3PS_4, including the results in Gupta et al. (Ref. 7), which also appears to show a site distortion/elongation in the average Na^+ probability density along directions of mobility. Other theory studies (see works by Mo, Wood, & Hautier, e.g.: 10.1021/acs.chemrev.9b00747, 10.1098/rsta.2019.0467, 10.1016/j.chempr.2019.07.001) have also highlighted that local site distortion tendency is often related to superionic behavior.

3) In light of the differences between Na_3PS_4 and Na_3PSe_4 (including the nature of the cation-anion anharmonic coupling), can the authors speculate on possible implications for understanding motivations for fast ion conduction in these two materials? Or on the relative transition temperatures between the two materials?

Reviewer: 2

Comments to the Author

In this manuscript, the authors reported THz-range Raman spectra of Na3PS4 and Na3PSe4, which were used to identify the anharmonic vibrational modes. DFT calculations were carried out to calculate the different phonon modes. While temperature-dependent Raman is traditionally used to identify phase transitions, the authors' results are thorough and the associated analyses are convincing. There is one question that the authors may consider addressing in the manuscript. How does the phase transition type (i.e. displacive and order/disorder) play a role in the superionic conductivity of this type of materials.

Author's Response to Peer Review Comments:

Omer Yaffe

Omer Yaffe
Chemical and Biological Physics
Weizmann Institute of Science
Rehovot, Israel
+97289343979
E-mail: omer.yaffe@weizmann.ac.il

May 18, 2022

To: Prof. Editor, Senior Editor, JPCL

Re: Revision for Manuscript ID jz-2022-00904c

Dear Prof. Editor,

On behalf of all the authors, I would like to thank the referees for their effort and comments that helped us improve this manuscript. In the revised manuscript we address all the comments and suggestions (see below the point-by-point response).

We are confident that our manuscript is now ready for publication in *J.Phys.Chem.Lett.*

Sincerely,

Omer Yaffe, Ph.D.

This response letter includes segments of the revised manuscript and supporting information (marked in red). For technical reasons we removed the citations from these segments. They are included in the revised text.

Referee 1.1: The interpretation of elevated-temperature Raman spectra using zero-temperature DFT calculations is precarious, particularly for fast ion conductors. These materials have disordered cation configurations, yet the eigenvectors are associated with instantaneously idealized Wyckoff positions. The anharmonic changes to the phonon modes are also not well captured (unless molecular dynamics are used). Can the authors comment on the robustness of their conclusions in light of this?

We agree with the reviewer that 0 K, harmonic calculations can be problematic for the study of ion conductors. However, we would like to stress that first, we have focused on the parent compounds which have fully occupied Na^+ crystallographic sites and, hence, are less challenging for the calculations as far as ionic disorder is concerned. Second, while we fully agree that anharmonicity is not included in these calculations, the computations can still identify the relevant atomic motions by means of comparison to the low-temperature Raman data which are nearly harmonic, in order to identify which type of dynamic coupling appears at higher temperatures. An analysis of the precise atomic motion, including predictions of the associated Raman activities might indeed require finite-temperature, molecular dynamics simulations. Hence, we do think the reviewer is correct that these issues should be mentioned and discussed in the article. To account for this comment, we made the following changes in the manuscript.

page 1, right col:

In a recent important study, Gupta *et al.* used neutron scattering and molecular dynamics (MD) to establish the connection between anharmonic phonon dynamics and ionic conductivity in Na_3PS_4 with a high concentration of Na^+ vacancies. Na_3PS_4 is the parent compound of $Na_{3-x}P_{1-x}W_xS_4$, a record Na^+ conductivity compound in which Na^+ vacancies have been introduced through tungsten doping.

page 2, right col:

First-principles calculations of the Raman spectra, based on density functional theory (DFT) and the harmonic approximation (see Methods), find a set of features similar to experiment for both compounds (see Fig. S1).

page 3, right col:

Keeping in mind that our 0 K phonon calculations do not account for any disorder or anharmoncity occurring at higher temperature, we can identify the soft modes in our DFT-computed Raman spectra of the low-temperature tetragonal phase (Fig. S1) by their frequency and symmetry.

Referee 1.2: The authors' interpretation seems to suggest that "nano" domains with different anharmonic tetragonal distortions persist in cubic Na3PS4, similar to dynamical averaging in perovskites. This is a very interesting conclusion, but it seems that another way to view these results is in terms of local cubic site distortions associated with a soft double-well potential in the cation potential energy landscape, which would manifest in the time-averaged cation probability density. Can the authors comment on this? It would be useful to more directly compare against MD studies of Na3PS4, including the results in Gupta et al. (Ref. 7), which also appears to show a site distortion/elongation in the average Na+ probability density along directions of mobility. Other theory studies (see works by Mo, Wood, Hautier, e.g.: 10.1021/acs.chemrev.9b00747, 10.1098/rsta.2019.0467, 10.1016/j.chempr.2019.07.001) have also highlighted that local site distortion tendency is often related to superionic behavior.

We fully agree with the referee and thank them for this comment. It was not our intention to suggest that the formation of static, nano domains is the only possible explanation

for our findings. To clarify this point we revised the manuscript accordingly.

page 1, left col:

The reduction of activation energy through chemical, structural, or dynamic frustration suggests shallow, strongly anharmonic energy landscapes in the lattice dynamics of the corresponding compounds. [Here we have added citations on local site distortion.]

page 5, left col:

Another route to understanding relaxational motion along this eigenvector is to picture the atoms involved - both mobile ions and host lattice - as sampling many configurations in a double-well potential along each of the crystallographic directions. Gupta et al. have established this mobile ion double well in Na_3PS_4 through nudged elastic band calculations.

Referee 1.3: In light of the differences between Na3PS4 and Na3PSe4 (including the nature of the cation-anion anharmonic coupling), can the authors speculate on possible implications for understanding motivations for fast ion conduction in these two materials? Or on the relative transition temperatures between the two materials?

Referee 2.1: There is one question that the authors may consider addressing in the manuscript. How does the phase transition type (i.e. displacive and order/disorder) play a role in the superionic conductivity of this type of materials.

We bundled these comments as they are very similar. We agree with both referee's and added the following discussion to the manuscript.

page 5, left col:

Since Na_3PS_4 displays a more extreme form of anharmonic lattice instability, it is reasonable to predict that it has the capacity to be a better ion conductor than Na_3PSe_4 under high Na^+ vacancy concentrations. However, the lower *t*-*c* transition temperature of Na_3PSe_4 suggests that it is easier for this lattice to shift lattice configurations, which could indicate the lattice is more amenable to ion hops when a high Na^+ vacancy concentration is present. An additional important factor is that the aliovalent doping and the correspondingly generated Na vacancies that give record high conductivity in these compounds can also affect qualitative changes to the behavior of this lattice instability, and this is an area that requires further research.
